# Effectiveness of health management team program to enhance prevention of mother-to-child transmission of hepatitis B virus in Ningxia, China

**DOI:** 10.1186/s12889-023-17550-2

**Published:** 2024-01-02

**Authors:** Chenglei Zhang, Yongxiang Huang, Liying Ji, Qian Zhu, Lixin Wang, Jingjiao Wang

**Affiliations:** 1 Medical Laboratory, General Hospital of Ningxia Medical University, Yinchuan, Ningxia 750003 China; 2 Department of laboratory, Yinchuan women and children healthcare hospital, Yinchuan, Ningxia 750001 China; 3 Department of Periodontics, Stomatological Hospital, General Hospital of Ningxia Medical University, 804 Shengli South Street, Xingqing District, Yinchuan, Ningxia 750003 China

**Keywords:** Hepatitis B, Mother-to-child transmission interruption, Health management team, Adverse outcomes

## Abstract

**Background:**

Hepatitis B mother-to-child transmission interruption (PMTCT) poses a formidable challenge in underdeveloped regions of China. This study aims to evaluate the effectiveness of PMTCT and the health management team (HMT) model in Ningxia, China, as well as the risk factors for adverse outcomes.

**Methods:**

The PMTCT + HMT model was established, and 360 pregnant women diagnosed with HBV infection in 2020–2022 were selected and divided into the control and the study groups based on different intervention modes. HBV serum markers and HBV DNA levels were assessed, the indicators of compliance behaviors and adverse outcomes were compared, and the factors influencing adverse outcomes were analyzed.

**Results:**

The majority of subjects were residents of the local city, married, with secondary school or higher education, and employees of public sectors. The proportion of ethnic minorities was 40.8% and 34.2% in the control group and study group. HBeAg positivity was 23.3% and 26.3%, and the proportion with HBV DNA levels ≥ 2 × 105 IU/mL was 9.2% and 7.1%. Compared with the control group (PMTCT alone), the PMTCT + HMT model led to improved maternal knowledge (17.5% vs. 57.1%), voluntary counseling (34.2% vs. 63.3%), and testing (37.5% vs. 70.4%). The incidence of adverse pregnancy outcomes ((including miscarriage, preterm birth) decreased significantly (17.5% vs. 6.2%), as did adverse neonatal outcomes (low birth weight and congenital HBV) (26.9% vs. 10.5%). Adverse outcomes were associated with low educational attainment, non-locals, unmarried status, and ethnic minority identity. Additionally, HBeAg positivity and HBV DNA levels ≥ 2 × 10^5^ IU/mL were risk factors for adverse outcomes.

**Conclusions:**

The PMTCT + HMT model demonstrates significant effectiveness in preventing mother-to-child transmission of hepatitis B in Ningxia. The unique demographic structure of Ningxia region is closely linked to poor outcomes, emphasizing the importance of monitoring HBeAg status and HBV DNA viral load level.

## Introduction

Globally, hepatitis B poses a serious public health challenge, leading to chronic and enduring adverse outcomes. Current data indicates approximately 296 million people worldwide are infected with Hepatitis B virus (HBV), 45% of which are located in the Western Pacific region [1, 2]. China, as a mid-to-high prevalence region, harbors over 90 million individuals with chronic hepatitis B, constituting roughly one-third of the global total [3]. Mother-to-child transmission (MTCT) remains a major source of chronic Hepatitis B infection, with transmission risk closely associated with the presence of Hepatitis B surface antigen (HBsAg), Hepatitis B e antigen (HBeAg), and high Hepatitis B DNA viral loads [4]. Without intervention, the rate of MTCT can be as high as 60-80% [2], leading to adverse pregnancy outcomes such as miscarriage and preterm labor, as well as adverse neonatal outcomes including low birth weight and neonatal deaths. Notably, approximately 25% of infected newborns may progress to chronic Hepatitis B, and in severe cases, develop cirrhosis and liver cancer [5]. This severity necessitates the adoption of more measures for Hepatitis B prevention and treatment, particularly concerning MTCT, to reduce adverse outcomes.

In view of the social and economic burdens posed by hepatitis B, the World Health Organization (WHO) has formulated the global Prevention of Mother-to-Child Transmission (PMTCT) plan, with the aim of eliminating Hepatitis B by 2030 and expanding Hepatitis B vaccine coverage to reduce MTCT rates actively [6–8]. China has responded positively to this global plan implementing a nationwide Expanded Program on Immunization (EPI), which includes prenatal screening, Hepatitis B vaccination, and Hepatitis B immunoglobulin (HBIG) injection. This initiative has yielded significant results, substantially reducing Hepatitis B infection rates among both adults and children [9, 10]. However, the problem is not entirely resolved, as the maintenance of MTCT rates still results in approximately 50,000 new Hepatitis B infections in newborns annually, directly correlated with the high prevalence of maternal infection, especially among mothers who are HBsAg or HBeAg positive [11]. Furthermore, the imperfect Hepatitis B MTCT system has hindered the implementation of the plan, with limited awareness, financial barriers to testing and diagnosis, and supply constraints for vaccines and treatment drugs impeding progress toward Hepatitis B elimination goals [12, 13].

Ningxia Hui Autonomous Region, as an inland province in the northwestern region of China, faces multiple constraints due to geographical, economic, and healthcare factors. These limitations have affected the application of the Hepatitis B PMTCT model and subsequently influenced its efficacy. Therefore, this study, building upon the PMTCT model within certain boundaries, fully leverages the advantages of healthcare institutions and introduces the concept of a Health Management Team (HMT), with the aim of exploring how to enhance health education, provide personalized interventions and monitoring measures in order to fill potential gaps in PMTCT model implementation. The objective is to validate the effectiveness of the PMTCT + HMT model in preventing mother-to-child transmission of hepatitis B, with the ultimate goal of minimizing Hepatitis B transmission and its adverse consequences. Furthermore, this study will pay special attention to the unique social and demographic characteristics of the region, conducting in-depth research to examine the relationship between these characteristics and the effectiveness of transmission prevention. This will facilitate the development of more precise strategies for Hepatitis B mother-to-child transmission prevention, providing scientific evidence and practical guidance to improve Hepatitis B prevention and control efforts in the region.

## Materials and methods

### Research objectives

This study, approved by the Ethics Committee of Ningxia Medical University General Hospital (Approval No.: 2018 − 367), obtained informed consent from all study subjects. The study subjects comprised 360 pregnant women who underwent prenatal examinations and were diagnosed with Hepatitis B virus (HBV) infection at Ningxia Medical University General Hospital and Yinchuan Maternal and Child Health Hospital between January 2020 and December 2022. They were randomly divided into control and study groups, and demographic data were collected.

### Methods

#### Establishment of PMTCT + HMT model

The PMTCT model was based on the fundamental strategy of “prevention first, effective monitoring, early diagnosis, and standardized management” and introduced the concept of the Health Management Team (HMT). The HMT comprised medical professionals from various hospital departments. Before intervening with the study subjects, team members received intensive training on the pathogenesis, transmission routes, prevention, and treatment measures of Hepatitis B, and tailored health management processes, content, and methods according to real-world conditions. This was done to enhance team members’ theoretical knowledge, educational skills, and management capabilities.

#### Intervention programs

Strict adherence to the “Clinical Management Process for Preventing Mother-to-Child Transmission of Hepatitis B Virus (HBV) (2021)” [14] was followed for the intervention of study subjects. The intervention protocol included: (a) Screening: Collecting fasting venous blood, separating serum, and quantitatively detecting HBV serological markers, including HBsAg and anti-HBs; (b) Disease assessment and treatment: Pregnant women testing positive for HBsAg underwent further evaluation, including HBeAg, anti-HBe, HBV DNA levels, liver function biochemical indicators, and ultrasound examinations, followed by appropriate antiviral treatment; (c) Antiviral treatment for MTCT prevention: Pregnant women with high HBV viral loads in the late stages of pregnancy received antiviral treatment based on their HBV DNA levels, along with vaccination and HBIG injection for newborns to reduce the risk of MTCT. (d) Neonatal immunization: Newborns were vaccinated against Hepatitis B or received combined Hepatitis B vaccination and HBIG immunization within 12 h after birth. Subsequently, planned immunizations were administered at different time points based on the newborn’s condition. In addition to this, the study group implemented a system where HMT members were responsible for health education for pregnant women, emphasizing the dangers of Hepatitis B and MTCT, providing testing and treatment plans, and encouraging their spouses to undergo serological testing. HMT members also provided comprehensive management, monitored indicator changes, followed standardized management procedures, and offered necessary psychological interventions.

#### Observational indicators

These included health knowledge acquisition, voluntary counseling rates, frequency of testing, and satisfaction rates for both groups. Furthermore, adverse pregnancy outcomes, neonatal adverse outcomes, and other parameters were monitored and compared between the two groups.

### Statistical analysis

Descriptive statistics were used for continuous data, presented as means and standard deviations (x ± s), while categorical data were described as frequencies and percentages [n (%)]. Between-group comparisons were conducted using Student’s t-test for continuous data and the chi-squared test for categorical data. Additionally, the odds ratio (OR) and 95% confidence interval (CI) were utilized to assess factors associated with adverse outcomes. All data analyses were performed using SPSS 18.0 software, with statistical significance set at *P* < 0.05.

## Results

### Basic characteristics of subjects

Firstly, we summarized and analyzed the basic demographic characteristics of the control group (120 individuals) and the study group (240 individuals) (Table 1). The average age of the study subjects in both groups was 30.52 years and 29.90 years, with the majority being residents of the local city (56.7% vs. 62.9%), married (88.3% vs. 90.8%), having attained at least a high school education (85.0% vs. 86.3%), predominantly employed in public service occupations (45.8% vs. 49.2%), and a proportion of ethnic minorities of 40.8% and 34.2%. No significant differences in these demographic characteristics were observed between the two groups. Additionally, we also paid attention to Hepatitis B-related indicators. A minority of pregnant women had a history of prior Hepatitis B virus infection (26.7% vs. 32.9%), and some had undergone treatment (24.2% vs. 32.5%). The initial serological test results during the course of this study showed that a portion of pregnant women tested positive for HBeAg (23.3% vs. 26.3%), while most had negative HBV DNA loads, with only 9.2% and 7.1% having levels ≥ 2 × 10^5^ IU/mL. These differences in test indicators were not statistically significant.

**Table 1:** Comparison of demographic characteristics and serological markers in subjects (n = 360)

	Control group	Study group	t/χ2	*P* value
Age (yrs)	30.52 ± 4.68	29.90 ± 4.63	1.393	0.234
Residency				
Locals	68(56.7)	151(62.9)	1.312	0.252
Non-locals	52(43.3)	89(37.1)		
Occupation				
Public employees	55(45.8)	118(49.2)	0.538	0.764
Business persons	42(35.0)	75(31.3)		
Others	23(19.2)	47(19.5)		
Education background				
Primary school	14(11.7)	26(10.8)	0.963	0.5801
Secondary school	73(60.8)	132(55.0)		
College or above	29(24.2)	75(31.3)		
Unknown	4(3.3)	7(2.9)		
Ethnicity				
Hui	49(40.8)	82(34.2)	1.536	0.215
Han	71(59.2)	158(65.8)		
Marital status				
Married	106(88.3)	218(90.8)	1.536	0.215
Unmarried	14(11.7)	22(9.2)		
Previous infection	32(26.7)	79(32.9)	1.465	0.226
Previous treatments	29(24.2)	78(32.5)	2.660	0.103
HBeAg positivity	28(23.3)	63(26.3)	1.063	0.548
HBV DNA (IU/mL)				
< 100	93(77.5)	177(73.7)	1.177	0.337
100-200000	16(13.3)	46(19.2)		
≥ 200000	11(9.2)	17(7.1)		

### Compliance behaviors

Through a survey questionnaire, an assessment of knowledge acquisition and satisfaction was conducted among study subjects, and differences in voluntary counseling rates and the frequency of testing were recorded between the two groups. The results revealed that in the control group, only 21 individuals (17.5%) had good knowledge acquisition, while conversely, 46 individuals (38.3%) had relatively poor knowledge acquisition. Additionally, the voluntary counseling rate in the control group was 34.2%. Consequently, only 37.5% of pregnant women in the control group underwent ≥ 3 serological marker tests during their pregnancy, with a dissatisfaction rate of 14.2%. In contrast, the study group exhibited a significant improvement in knowledge acquisition, with a good acquisition rate of 57.1% and a reduced rate of poor knowledge acquisition at 10.8%. Moreover, the voluntary counseling rate significantly increased to 63.3%, and 70.4% of pregnant women had undergone ≥ 3 testing sessions. The overall dissatisfaction rate decreased to only 2.1% in the study group. These differences were statistically significant when compared to the control group (Table 2).

**Table 2:** Compliance behaviors of subjects in the two groups (n = 360)

	Control group	Study group	t/χ2	*P* value
Knowledge/awareness				
Good	21 (17.5)	137 (57.1)	62.04	< 0.001
Average	53 (44.2)	77 (32.1)		
Poor	46 (38.3)	26 (10.8)		
Voluntary counseling	41 (34.2)	152 (63.3)	27.36	< 0.001
Number of tests for Hepatitis B				
≥ 3	45 (37.5)	169 (70.4)	50.38	< 0.001
2	49 (40.8)	64 (26.7)		
1	26 (21.7)	7 (2.9)		
Satisfaction				
Satisfied	40 (33.3)	142 (59.2)	33.16	< 0.001
Somewhat satisfied	63 (52.5)	93 (38.7)		
Dissatisfied	17 (14.2)	5 (2.1)		

### Adverse outcomes

Among the 120 study subjects in the control group, there were 5 cases of miscarriage and 16 cases of premature birth, resulting in an adverse pregnancy outcome rate of 17.5%. Additionally, there were 19 cases of low birth weight and 12 cases of congenital hepatitis B, leading to a neonatal adverse outcome rate of 26.9%. In contrast, among the 240 study subjects in the study group, only 1 case of miscarriage and 14 cases of premature birth occurred, with 16 cases of low birth weight and 9 cases of congenital hepatitis B. The rates of adverse pregnancy outcomes (6.2%) and neonatal adverse outcomes (10.5%) were significantly lower in the study group compared to the control group, with statistically significant differences observed (Table 3).

**Table 3:** Comparison of adverse outcomes in subjects (n = 360)

	Control group	Study group	χ2	*P* value
Adverse pregnancy outcomes				
Miscarriage	5 (4.2)	1(0.4)	6.86	0.0088
Preterm labor	16 (13.3)	14 (5.8)	5.89	0.0152
Adverse neonatal outcomes				
Low-birth-weight babies	19 (16.5)	16 (6.7)	7.66	0.0056
Congenital Hepatitis B	12 (10.4)	9 (3.8)	5.69	0.0171

### Factors associated with adverse outcomes

As shown in Table 4, among the study subjects, urban residents had a lower probability of experiencing adverse pregnancy outcomes and neonatal adverse outcomes (OR = 0.42 and OR = 0.58, respectively), while those with higher educational attainment (OR = 0.23) and those employed in public service occupations (OR = 0.51) were relatively less likely to experience adverse outcomes. However, pregnant women belonging to ethnic minorities had a higher likelihood of adverse outcomes (OR = 3.30), although this risk was reduced in the study group (OR = 2.49). Additionally, unmarried status was associated with a higher probability of adverse outcomes (OR = 4.36), with a decreasing trend observed after intervention in the study group (OR = 3.34). Furthermore, it was observed that HBeAg positivity (OR = 15.07) and HBV DNA levels ≥ 2 × 105 IU/mL (OR = 27.59) were positively correlated with the probability of adverse outcomes.

**Table 4:** Factors associated with adverse outcomes (n = 360)

	AO* Control/Study Group N(%)	Control group		Study group	
		OR (95% CI)	*P* value	OR (95% CI)	*P* value
Residency					
Locals	17(14.2)/14(5.8)	0.42(0.21–0.91)	< 0.05	0.58(0.26–1.17)	0.132
Non-locals	23(19.2)/14(5.8)	1		1	
Occupation					
Public employees	14(11.7)/13(5.4)	0.51(0.28–1.11)	0.092	0.88(0.41–1.88)	0.758
Business persons and Others	26(21.7)/15(6.3)	1		1	
Education background					
College or above	4(3.3)/7(2.9)	0.23(0.08–0.65)	< 0.05	0.67(0.25–1.68)	0.385
Primary and Secondary school	36(30.0)/21(8.8)	1		1	
Ethnicity					
Hui	24(20.0)/15(6.3)	3.30(1.50–7.09)	< 0.05	2.49(1.17–5.49)	< 0.05
Han	16(13.3)/13(5.4)	1		1	
Marital status					
Unmarried	9(7.5)/6(2.5)	4.36(1.32–12.32)	< 0.05	3.34(1.17–9.11)	< 0.05
Married	31(25.8)/22(9.2)	1		1	
HBeAg					
positive	22(18.3)/19(7.9)	15.07(5.10-42.74)	< 0.05	8.06(3.31-19.00)	< 0.05
negative	18(15.0)/9(3.8)	1		1	
HBV DNA (IU/mL)					
≥ 2 × 10^5^	10(8.3)/10(4.2)	27.59(4.21–302.9)	< 0.05	16.27(5.71–43.77)	< 0.05
< 2 × 10^5^	30(25.0)/18(7.5)	1		1	

*AO: adverse outcomes

## Discussion

Facing the severe epidemic situation of hepatitis B, the Chinese government has actively responded to the World Health Organization’s call and implemented effective measures, including expanding prenatal screening, promoting timely vaccination, and standardizing antiviral treatment. These measures have successfully reduced the risk of mother-to-child transmission of hepatitis B. This initiative not only decreases the probability of infant infection but also reduces the risk of developing chronic hepatitis B, leading to significant economic benefits. Research data indicates that the PMTCT model saves 7,726.03 dollars per Quality-Adjusted Life Year (QALY), making it a cost-effective strategy [15, 16]. Therefore, relatively underdeveloped countries or regions, especially those with a significant minority population like Ningxia, with nearly 36% of the population belonging to minority groups and relatively lagging economies, should prioritize the implementation of the hepatitis B PMTCT model.

However, in reality, the implementation of the PMTCT model lags significantly behind the plan. Imperfections in the social and healthcare systems have resulted in various issues, including low health awareness among the population, unequal resource distribution, challenges in achieving universal screening, and process gaps. For example, globally, many HBV-infected individuals are unaware or do not prioritize their condition [17], indicating insufficient health education and limited screening. This study also found that the historical HBV infection rate among pregnant women is only about 30.0%, with most of them unaware of their infection during pregnancy or a past period, leading to missed interventions. To address these issues, this study introduced the Health Management Team (HMT) method on top of the PMTCT model, with an organizational structure similar to Liberia’s health management plan but more targeted [18]. Comprising healthcare professionals from various institutions and fields, the HMT, through clear division of labor and efficient deployment, streamlined the workflow, further standardized the processes, and enhanced management oversight, ensuring the effective application of the PMTCT model. Through effective health education, pregnant women’s disease knowledge significantly improved, with a rise in the proportion of those with good knowledge from 17.5 to 57.1%. Additionally, voluntary counseling rates increased on the foundation of basic disease knowledge, leading to improved doctor-patient communication and closer relationships. This helps ensure an adequate number of serological marker tests during pregnancy and significantly enhances pregnant women’s satisfaction. Furthermore, HMT members’ professional skills and teamwork abilities were further enhanced.

Under the comprehensive application of the PMTCT + HMT model, the probability of adverse pregnancy outcomes significantly decreased, and the blocking effect on congenital hepatitis B in newborns became evident. Although there is still some way to go to achieve the goal of controlling mother-to-child transmission rates to below 2% [19] and reach the Western Pacific Region’s target of a childhood hepatitis B prevalence of no more than 0.1% by 2030 [7], our research clearly demonstrates the feasibility of the PMTCT + HMT model. It confirms that taking timely measures to address deficiencies in preventing mother-to-child transmission of hepatitis B by 2030 is a positive and beneficial approach. In terms of sociodemographic characteristics, pregnant women with a university education or higher and those residing in the city had a lower likelihood of adverse outcomes, likely due to better job opportunities and access to healthcare resources and services [20]. Being unmarried was considered a favorable factor for adverse outcomes, possibly because unmarried individuals have more opportunities for unknown sexual partners with HBV infection, a finding consistent with a study in Ethiopia [21]. However, other research has suggested the opposite, indicating that married pregnant women are more likely to be infected with HBV [22], and these differences may depend on the country, geographic location, and cultural background of the study population. Minority groups also showed a trend toward adverse outcomes, possibly related to the unique religious and cultural practices of the Hui minority created by the local Hui autonomous system. Additionally, consistent with multiple research results [23, 24], HBeAg positivity and HBV DNA levels ≥ 2 × 105 were risk factors for adverse outcomes and a major reason for possible PMTCT failure. HBeAg is a soluble small antigen that may induce immune tolerance in newborns through the placental barrier [25], and HBeAg positivity is usually highly correlated with high levels of HBV DNA replication, which may increase the risk of mother-to-child transmission [26]. Therefore, prioritizing the detection of HBeAg status and HBV DNA viral load levels is essential for timely antiviral therapy to block mother-to-child transmission.

The strengths of this study lie in validating the effectiveness of the PMTCT + HMT model in interrupting mother-to-child transmission of hepatitis B in underdeveloped inland regions of China and proposing solutions to existing problems. However, the study has some potential limitations. Firstly, due to resource constraints and the COVID-19 pandemic, data collection was limited in terms of geographical range and time, and follow-up in childhood, serological marker monitoring, and outcome evaluation have not been fully implemented. Nevertheless, these limitations are unlikely to significantly affect the assessment of model effectiveness. Secondly, this study did not include pregnant women who were not infected with the hepatitis B virus, lacking baseline data for comparison, necessitating further research to understand the occurrence rate of adverse outcomes among uninfected pregnant women. Future research needs to expand the sample size, overcome spatial and temporal limitations to comprehensively evaluate the effectiveness of the PMTCT model in hepatitis B prevention in this region. However, anticipated challenges include the limited availability of HBV DNA test results for pregnant women, which may hinder large-scale investigations and the promotion of the PMTCT model. Despite HBeAg testing being readily available, it cannot replace the role of HBV DNA in disease assessment and treatment [27–28]. Therefore, developing a rapid and cost-effective HBV DNA testing method is necessary and a crucial technological focus in the field of PMTCT research, vital for achieving the 2030 elimination goals.

## Conclusion

The PMTCT + HMT model has shown significant effectiveness in interrupting mother-to-child transmission of hepatitis B in the Ningxia region and should be actively promoted and continuously improved to ensure its effective operation. Additionally, the unique population structure in Ningxia is closely related to adverse outcomes of hepatitis B, and it requires comprehensive consideration based on actual conditions. Emphasis should be placed on two critical factors, HBeAg status and HBV DNA viral load levels, to better address this health challenge.

## Data Availability

The data that support the findings of this study are available from the corresponding author upon reasonable request.
